# Posterior Cruciate Ligament Repair With Suture Augmentation: A Report of Two Cases With Two-Year Follow-Up

**DOI:** 10.7759/cureus.12447

**Published:** 2021-01-03

**Authors:** Henry T Shu, Paolo Rigor, Brian J Panish, Patrick Connolly, Evan Argintar

**Affiliations:** 1 Orthopaedic Surgery, Johns Hopkins University School of Medicine, Baltimore, USA; 2 Orthopaedic Surgery, Georgetown University School of Medicine, Washington DC, USA; 3 Orthopaedic Surgery, Washington Hospital Center, Washington DC, USA

**Keywords:** knee dislocation, posterior cruciate ligament, ligament repair, multiligamentous knee injury, internal brace

## Abstract

We present two cases of posterior cruciate ligament (PCL) repair with suture augmentation (SA) in the setting of multiligamentous knee injury (MLKI). Excellent clinical outcomes were obtained at two-year follow-up with both patients returning to sport following injury. Both patients demonstrated improvements in Knee Injury and Osteoarthritis Outcome Score (KOOS) that exceeded the minimal clinically important difference (MCID) as reported in the literature for ligamentous knee injuries. One patient developed arthrofibrosis, which was successfully treated with manipulation under anesthesia and arthroscopic lysis of adhesions two months postoperatively. Both patients had full knee range of motion (ROM) by a one-year follow-up. One patient returned to full preinjury level of sport at six months postoperatively while the other patient returned to 50% of preinjury intensity at two-year follow-up. This series of two cases of PCL repair with SA in MLKIs demonstrates that PCL repair with SA is a viable procedure that can result in excellent short-term outcomes and restore knee stability.

## Introduction

While isolated posterior cruciate ligament (PCL) injuries are rare, they commonly occur in the context of multiligamentous knee injuries (MLKIs) [1]. Historically, open repair for PCL injuries was largely replaced by arthroscopic PCL reconstruction due to poor outcomes [2-6]. Recently, there has been a renewed interest in arthroscopic PCL repair as a viable treatment option for proximal and distal PCL tears [7]. There remains a paucity of literature discussing PCL repair outcomes. There are currently no studies reporting patient-reported outcome measures (PROMs) for PCL repair with suture augmentation (SA) in either isolated injuries or in the setting of MLKIs. We reviewed all cases of PCL repair with SA performed by a single surgeon at our institution from April 2013 up until April 2018 and identified two cases. This case series describes both patients who underwent PCL repair with SA in the setting of MLKIs following a knee dislocation. Both patients were informed that data concerning their cases would be submitted for publication, and both patients provided informed consent.

## Case presentation

Case 1

A 19-year-old male was playing basketball when his left knee dislocated after jumping and landing on his left leg. He was taken to a nearby hospital where his left knee was subsequently reduced and immobilized. He was then referred to our institution.

**Figure 1:**
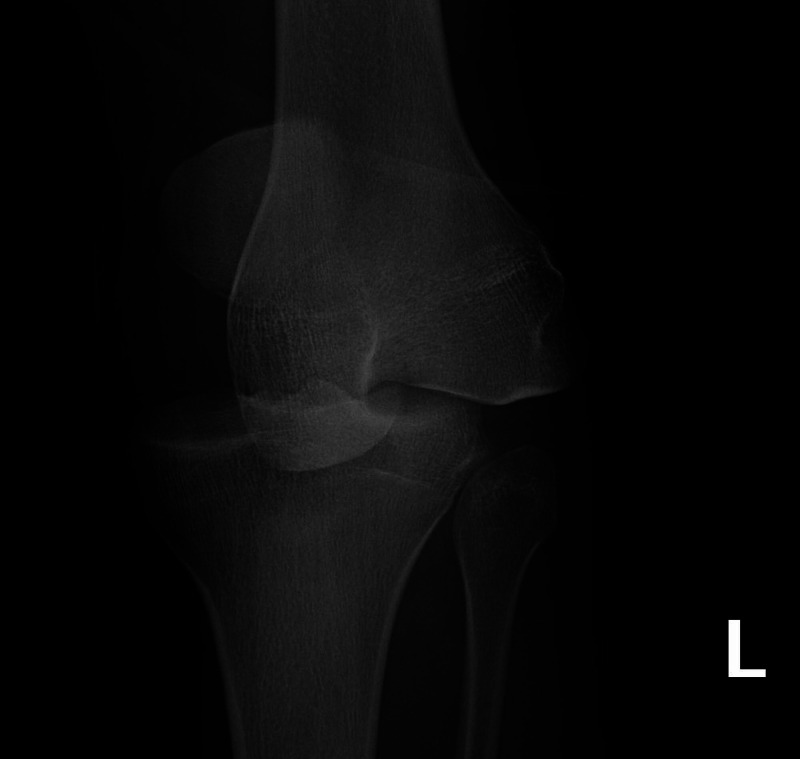
Anterior–posterior radiograph of the left knee in case 1 Anterior–posterior radiograph of the left knee demonstrating acute knee dislocation.

His initial examination demonstrated diffuse knee swelling, severe varus laxity, and moderate valgus laxity at 30 degrees of flexion. An anterior-posterior radiograph demonstrated an acute knee dislocation (Figure 1). Lateral radiographs of this patient, which demonstrated an anterior knee dislocation, were not stored in the electronic medical records system as they were initially taken at an outside hospital.

**Figure 2:**
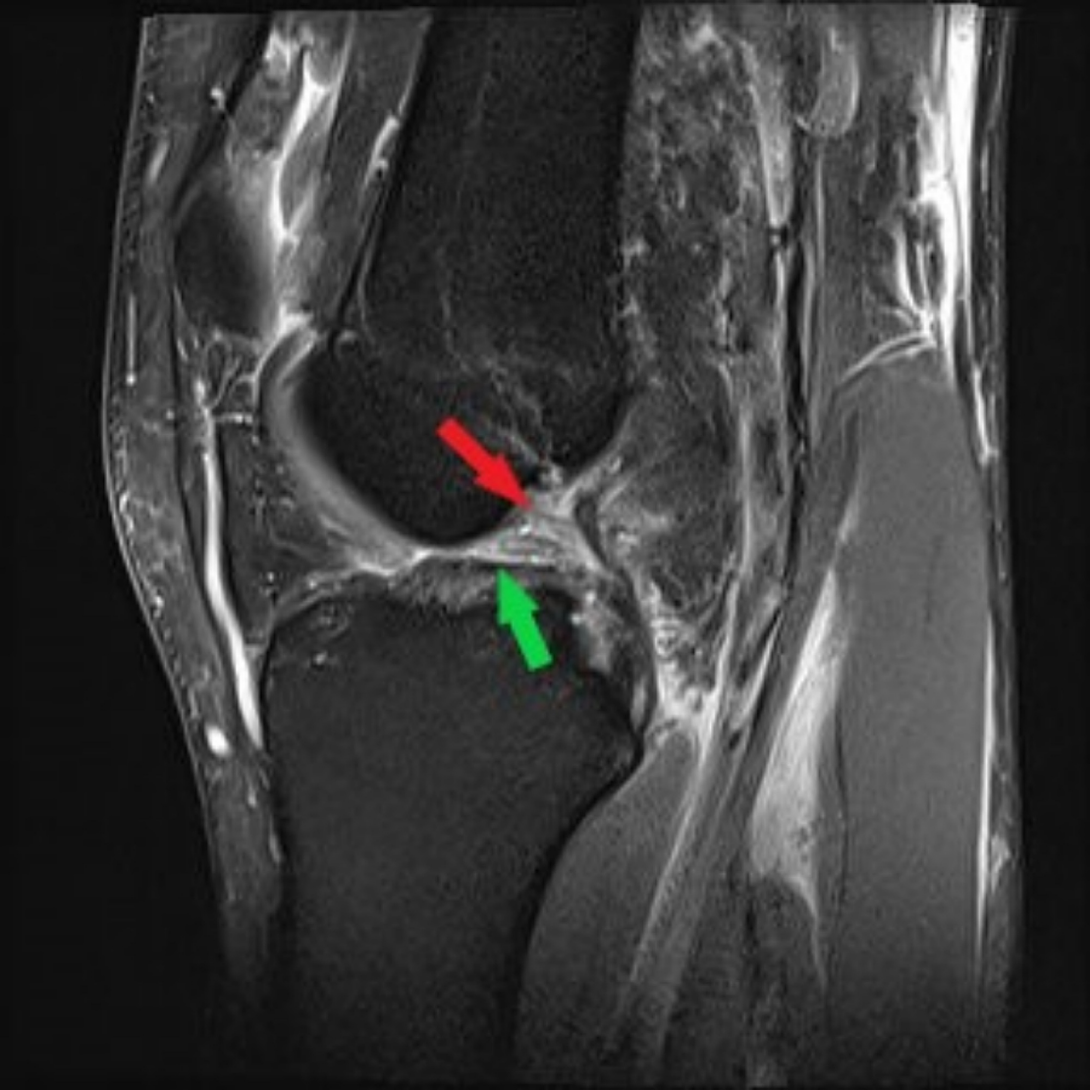
Sagittal T2-weighted MRI of the left knee in patient 1 Sagittal T2-weighted MRI of the left knee demonstrating complete disruption of the ACL (green arrow) and proximal detachment of the PCL (red arrow).

**Figure 3:**
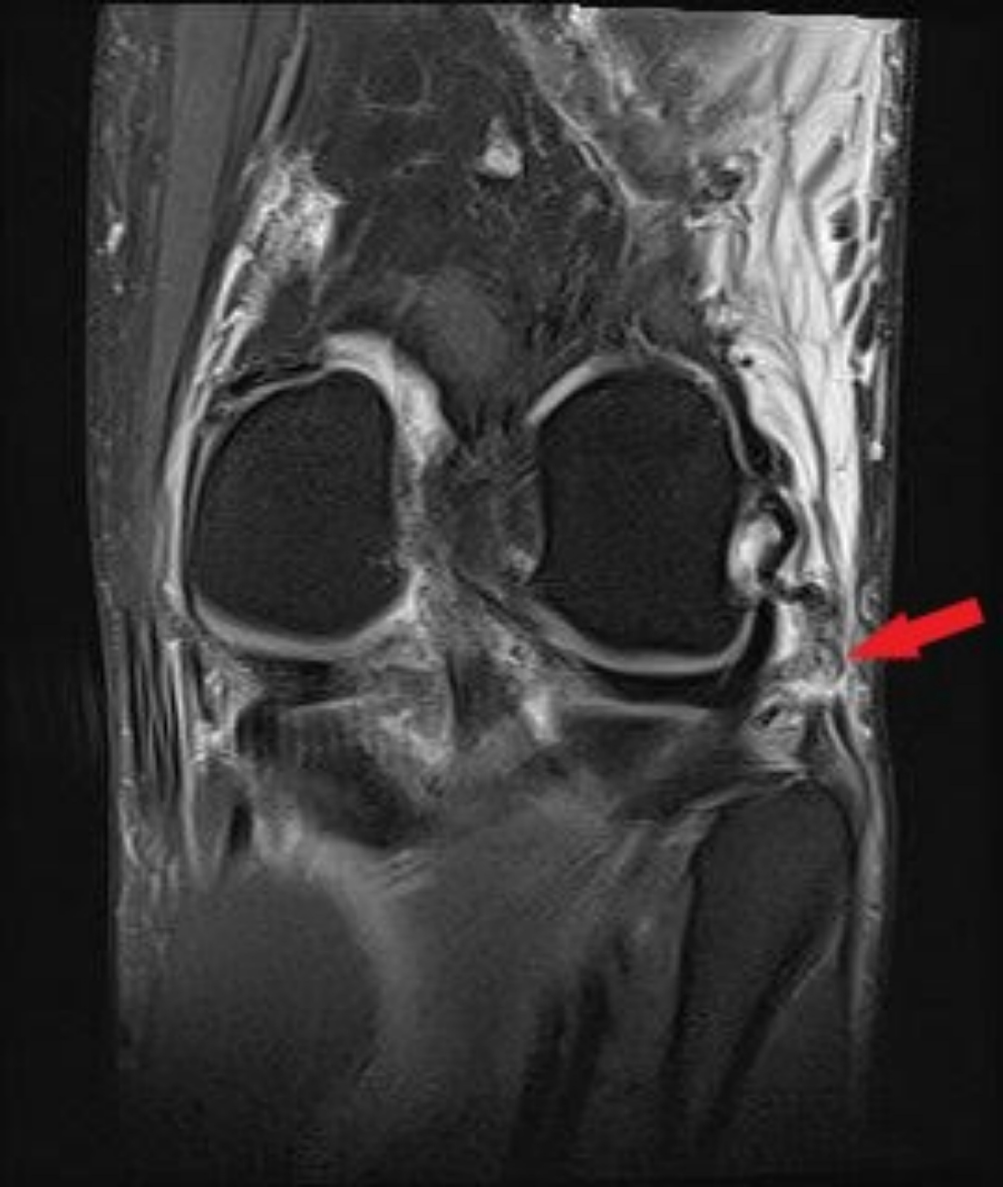
Coronal T2-weighted MRI of the left knee in patient 1 Coronal T2-weighted MRI of the left knee demonstrating LCL tear (red arrow).

He was neurovascularly intact distally. He demonstrated a positive Lachman test and a positive posterior drawer. Magnetic resonance imaging (MRI) demonstrated complete rupture of the anterior cruciate ligament (ACL), grade two sprain of the PCL proximally, grade two sprain of the medial collateral ligament (MCL), complete rupture of the biceps femoris attachment and lateral collateral ligament (LCL), an increased signal in the lateral meniscus, and fraying of the posterior root of the medial meniscus (Figures 2 and 3).

Surgical technique

**Figure 4:**
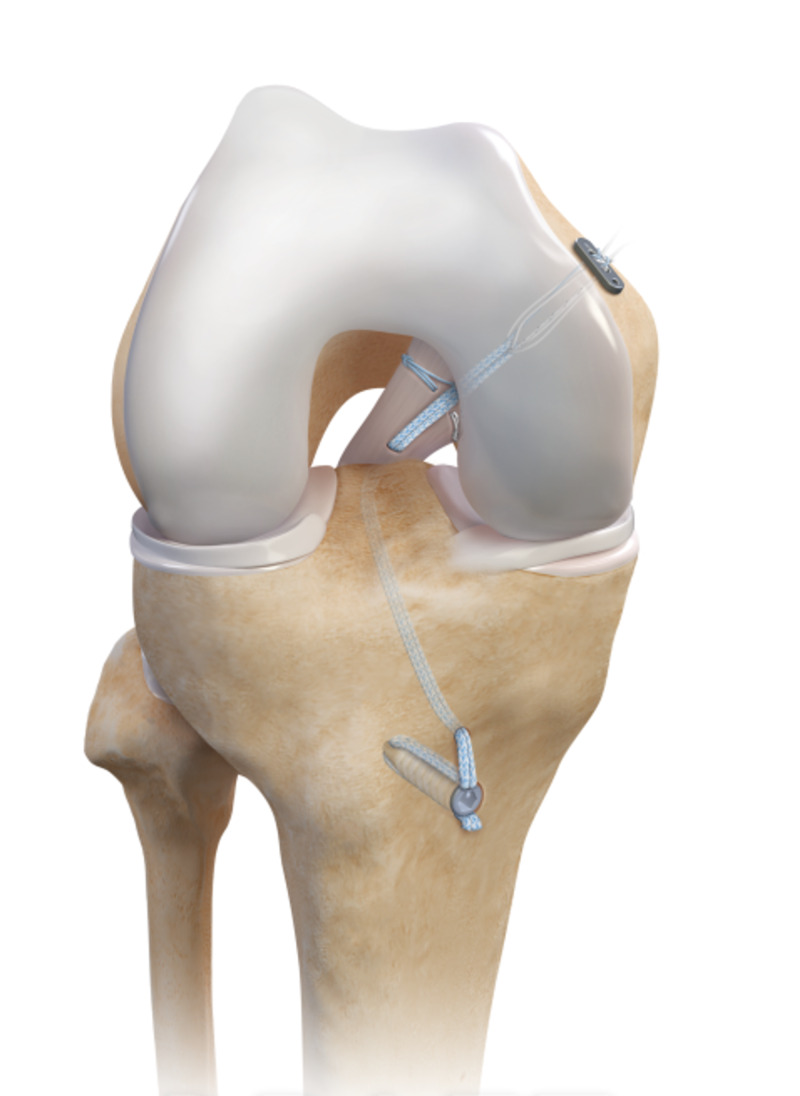
Final PCL repair construct with SA Final PCL repair constructs with SA (Internal Brace, Arthrex, Inc., Naples, FL). The PCL was repaired with sutures (FiberWire and TigerWire, Arthrex, Inc., Naples, FL) in a luggage-tag fashion. The SA and PCL were fixed proximally with suspensory fixation (TightRope, Arthrex, Inc., Naples, FL), and a button (External Attachable Button System, Arthrex, Inc., Naples, FL). The SA was fixed distally with a 4.75 mm biocomposite bone anchor (4.75 mm Swivelock, Arthrex, Inc., Naples, FL).

Following informed consent, the patient underwent surgery three days postinjury. The lower extremity was prepped and draped with standard, sterile technique. Initial examination under anesthesia (EUA) demonstrated a full range of motion (ROM), positive anterior drawer, Lachman, pivot shift, and varus opening. He also had a positive posterior drawer test and a positive posterior sag test. The PCL injuries, including the LCL and biceps femoris rupture, were repaired first through a posterolateral curvilinear incision. Then, anterolateral and anteromedial arthroscopic portals were created, and diagnostic arthroscopy was performed. The PCL demonstrated a near full-thickness tear at its proximal origin. The ACL was also fully torn, and the lateral meniscus had a vertical-type tear that opened to probing. The medial meniscus was not torn. Repair of the PCL started with debridement of its proximal attachment on the femur. The PCL was then mobilized and approximated to its origin with an anterior drawer force applied to the knee to confirm sufficient length for repair. The PCL was then sutured with alternating hitches in a luggage-tag manner using a labral suture passer and the sutures pulled through the anteromedial portal. An ACL drill guide was then used to create a femoral tunnel with a combined guide-pin retrograde reamer. Then, the retrograde reamer was used to ream 1 mm of the femoral cortex to stimulate bleeding and allow better healing of the PCL to the femur. Similarly, a tibial tunnel was drilled for fixation of the SA distally. A suspensory fixation construct was then created utilizing a button and the SA (Internal Brace, Arthrex, Inc., Naples, FL). The construct was then passed back into the knee, through the femoral tunnel, and the button flipped over the medial cortex of the medial femoral condyle with shuttling sutures. The SA was subsequently passed through the tibial tunnel and fixed to the tibia with a 4.75 mm biocomposite anchor. The PCL and SA were not tensioned yet, as all tensioning was done simultaneously following repair and/or reconstruction of all the injured ligaments. The final PCL repair construct can be seen in Figure 4.

The lateral meniscus was then repaired followed by ACL reconstruction with hamstring allograft and SA. The PCL and its SA were first tensioned with the knee in 90 degrees of flexion and an anterior drawer force applied. Then, the other constructs were tensioned. On completion, there was a negative anterior and posterior drawer, negative pivot shift, negative posterior sag, no varus opening, and full ROM.

Postoperative course

Postoperatively, the patient was non-weight bearing on his left knee for six weeks. His ROM was 5-50 degrees of flexion one week postoperatively and he initiated physical therapy four weeks postoperatively. At the four-month follow-up, the ROM was 0-125 degrees of flexion with grade 1 opening to varus. His final in-office follow-up at seven months demonstrated a full ROM, no abnormal knee laxity, and no evidence of tenderness or effusion. Throughout his postoperative course, his pain was well controlled and was adherent to all prescribed physical therapy.

A standardized questionnaire was administered preoperatively and at 24 months postoperatively to collect PROMs (Table 1). He stated that he was able to fully return to preinjury competitive activity level, which included playing pick-up basketball for up to five days a week, at six months postoperatively.

**Table 1:** PROMs of knee function of patient 1 at 24 months All patient-reported outcome measures (PROMs) were collected using a standardized questionnaire. Scores included Knee injury and Osteoarthritis Outcome Score (KOOS), Western Ontario and McMaster Universities Osteoarthritis Index (WOMAC), and Single Assessment Numeric Evaluation (SANE). The five KOOS subscales included: symptoms, pain, activities of daily living (ADL), sports and recreation (Sports and Rec), quality of life (QOL). Average pain scores were reported based on a (0–10) subjective rating scale. Return to play was reported based off a binary (Yes or No) response. If the patient responded Yes, time to RTP was reported. If the patient responded No, a percentage of baseline preinjury activity was reported.

	Preoperative	24 months postoperative (change from preoperative score)
KOOS	15.5	98.8 (83.3)
Symptoms	21.4	96.4 (75.0)
Pain	13.9	100.0 (86.1)
ADL	19.1	100.0 (80.9)
Sports and Rec	0.0	100.0 (100.0)
QOL	12.5	93.8 (81.3)
WOMAC	15.9	99.2 (83.3)
SANE	10.0	100.0 (90.0)
Average daily pain	7.0	0.0
RTP (Yes or No)	Yes, full RTP at six months post-op	

Case 2

**Figure 5:**
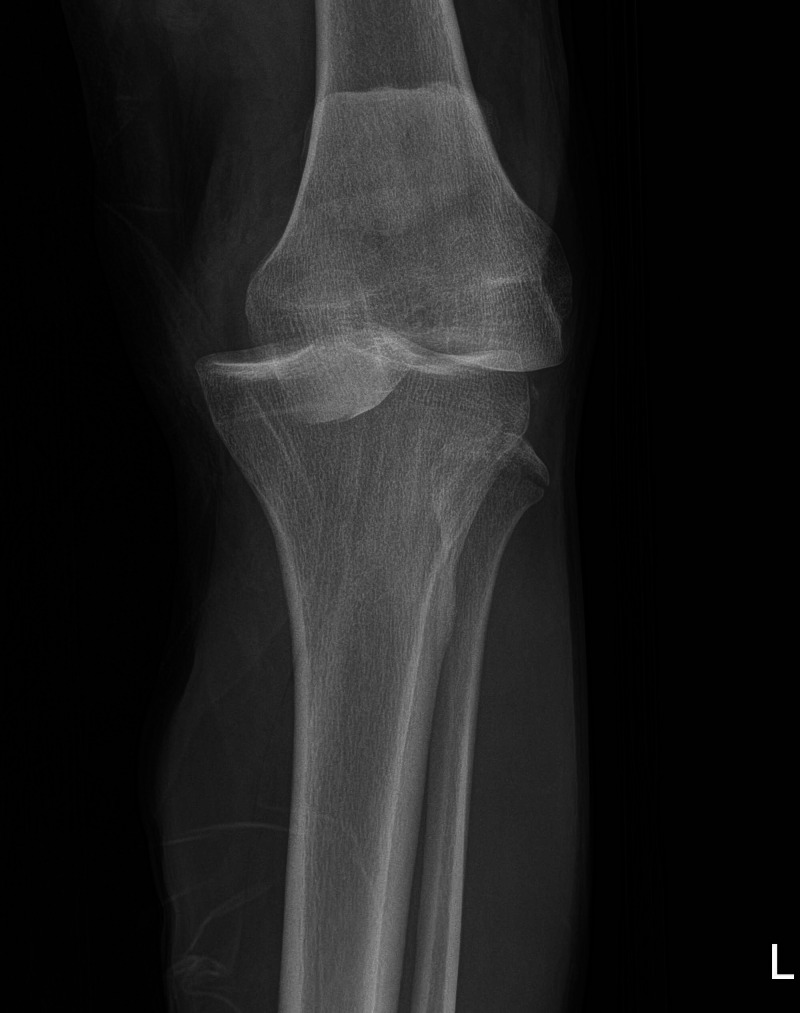
Anterior–posterior radiograph of the left knee in patient 2 Anterior–posterior radiograph of the left knee demonstrating acute knee dislocation.

**Figure 6:**
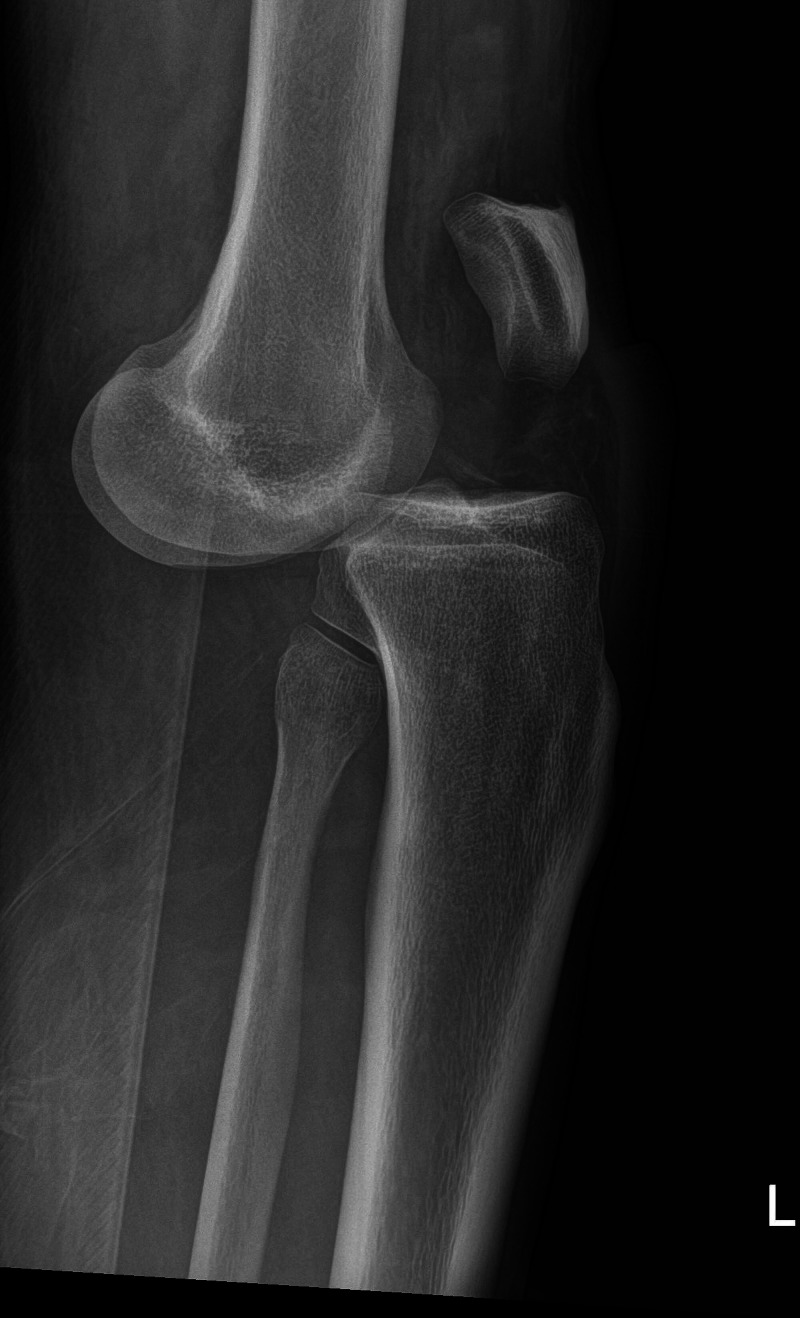
Lateral radiograph of the left knee in patient 2 Lateral radiograph of the left knee demonstrating acute anterior dislocation.

A 32-year-old male was presented to the emergency room with left knee deformity and pain after a motor vehicle accident. The patient was unable to provide a coherent history given phencyclidine intoxication. His initial left knee examination demonstrated anterior tibial dislocation, suggesting MLKI. ROM was deferred given gross instability. Radiographs demonstrated acute anterior dislocation (Figures 5 and 6). His reflexes were normal, and he was neurovascularly intact distally. His left knee was then reduced and placed in a knee immobilizer.

**Figure 7:**
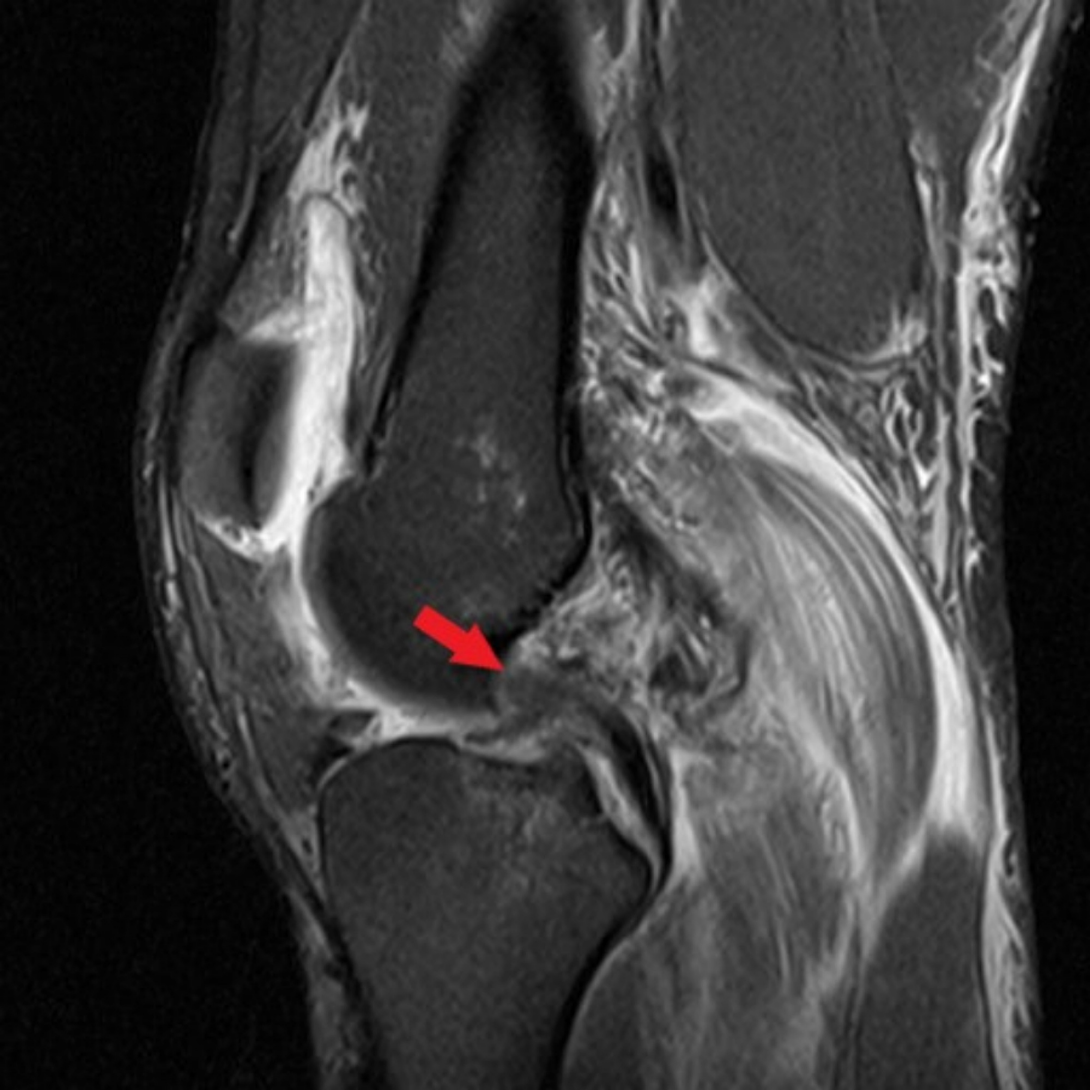
Sagittal T2-weighted MRI of the left knee in patient 2 Sagittal T2-weighted MRI of the left knee demonstrating an increased signal in the proximal aspect of the PCL (red arrow), suggesting possible PCL avulsion injury. This was supported by a physical examination and further confirmed intraoperatively.

**Figure 8:**
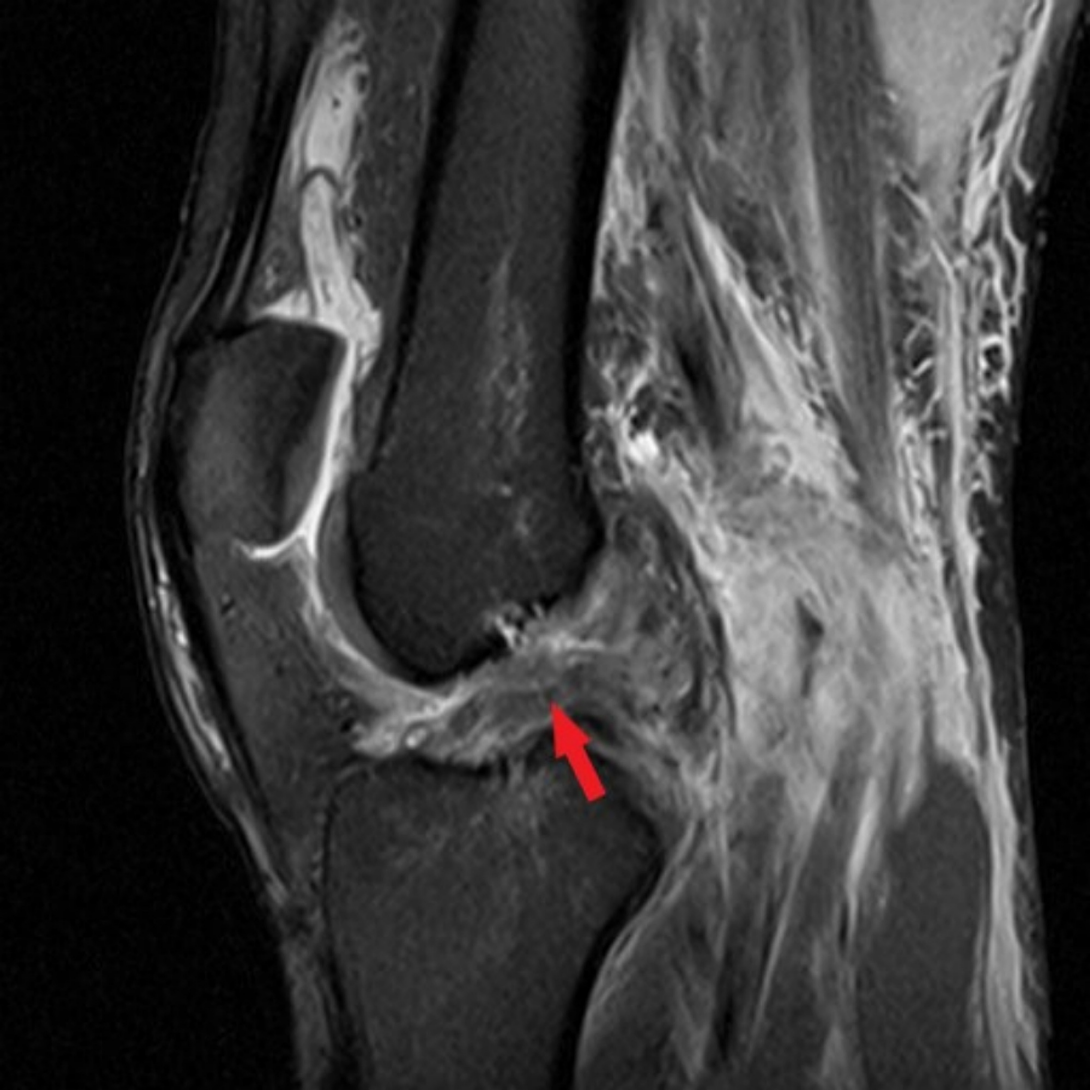
Sagittal T2-weighted MRI of the left knee in patient 2 Sagittal T2-weighted MRI of the left knee demonstrating complete rupture of the ACL (red arrow).

**Figure 9:**
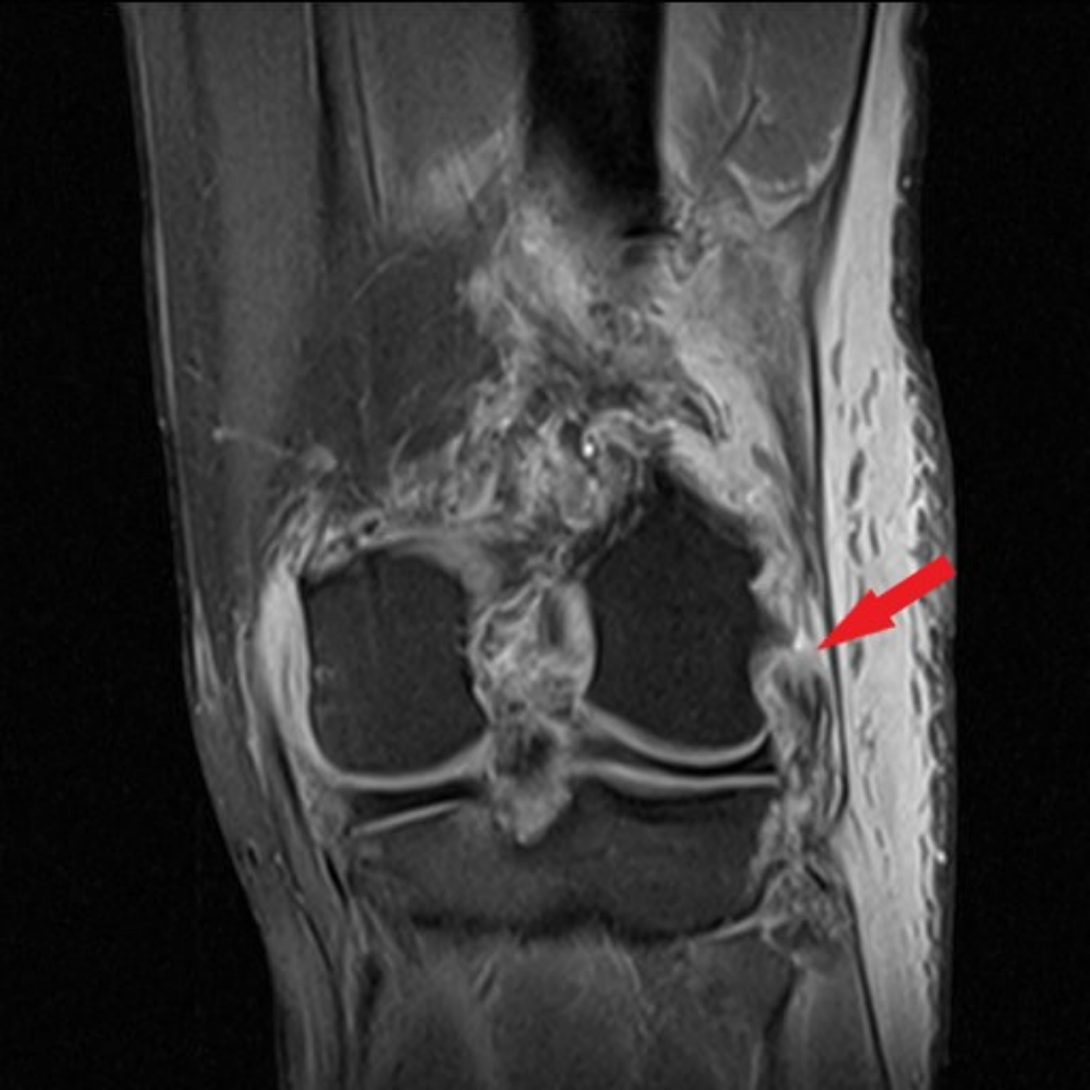
Coronal T2-weighted MRI of the left knee in patient 2 Coronal T2-weighted MRI of the left knee demonstrating rupture of the LCL (red arrow).

MRI later revealed a complete tear of the ACL, grades 2-3 sprain of the PCL proximally, grade 3 sprain of the MCL, grade 3 sprain of the LCL at its proximal insertion, fraying/low-grade strain of the popliteus tendon, and vertical linear signal extending between the posterior horn lateral meniscus and posterolateral capsule, suggesting meniscocapsular separation (Figures 7-9).

Surgical technique

After informed consent, the patient underwent surgery three days postinjury. Initial EUA revealed the absence of flexion contraction, full ROM, positive anterior drawer, positive pivot shift, positive posterior drawer, and positive posterior sag. The PCL was repaired in a similar fashion to patient 1 (Figure 4). Intraoperatively, the PCL was identified to be avulsed at its proximal insertion. Reconstruction of the ACL and LCL repair and lateral meniscus was also performed using similar techniques in case 1. Upon completion, there was a full ROM and no abnormal laxity.

Postoperative course

Postoperatively, the patient was instructed to be non-weight-bearing for four weeks. However, he followed-up in clinic three weeks postoperatively already partial weight-bearing on his left knee. He stated that he had been weight-bearing without a knee brace immediately postoperatively. At this point, his knee ROM was 0-20 degrees of flexion. He was advised to pursue an aggressive physical therapy regimen to improve ROM.

At two months postoperatively, the patient complained of continued stiffness in the left knee with ROM of 5-70 degrees of flexion. This prompted the patient to undergo manipulation under anesthesia and arthroscopic lysis of adhesions. At three months post-initial surgery, the patient was ambulating without difficulty. At his final in-office follow-up at 14 months, ROM was 0-130 degrees of flexion, no abnormal knee laxity, and no evidence of tenderness or effusion. Throughout his postoperative course, he was noncompliant with physical therapy. He stated that he was not able to return to full preinjury activity level and reports that he was able to perform baseline or preinjury activities at only 50% of preinjury intensity. Preoperative and 24-month postoperative PROMs are reported in Table 2.

**Table 2:** PROMs of knee function of patient 2 at 24 months All patient-reported outcome measures (PROMs) were collected using a standardized questionnaire. Scores included Knee Injury and Osteoarthritis Outcome Score (KOOS), Western Ontario and McMaster Universities Osteoarthritis Index (WOMAC), and Single Assessment Numeric Evaluation (SANE). The five KOOS subscales included: symptoms, pain, activities of daily living (ADL), sports and recreation (Sports and Rec), and quality of life (QOL). Average Pain scores were reported based on a (0–10) subjective rating scale. Return to Play was reported based off a binary (Yes or No) response. If the patient responded Yes, time to RTP was reported. If the patient responded No, a percentage of baseline preinjury activity was reported.

	Preoperative	24 months postoperative (change from preoperative score)
KOOS	4.8	78.0 (73.2)
Symptoms	28.6	67.9 (39.3)
Pain	0.0	97.2 (97.2)
ADL	0.0	94.1 (94.1)
Sports and Rec	0.0	25.0 (25.0)
QOL	0.0	50.0 (50)
WOMAC	6.1	88.7 (82.6)
SANE	0.0	60.0 (60.0)
Average daily pain	10.0	0.0
RTP (Yes or No)	No, can perform preinjury activities at only 50% of the preinjury intensity	

## Discussion

In our series of two cases, we demonstrate that PCL repair with SA is a viable technique for PCL repair in the setting of MLKIs. Both patients demonstrated excellent PROMs, with one patient returning to full preinjury activity level at six months and the other returning to about 50% of baseline activity level at two years. Although the second patient developed arthrofibrosis, he demonstrated full ROM without abnormal laxity at final in-clinic follow-up at 14 months. It is important to note that the second patient was non-compliant with weight-bearing restrictions and physical therapy. Both patients had changes in PROMs and ROM that exceeded minimum clinically important differences (MCIDs) as described in the current literature [8-10].

The first arthroscopic PCL repairs were described by Wheatley et al. [11]. In their case series, all repairs involved proximal PCL avulsion-type injuries, and 11 of their patients sustained MLKIs. They noted excellent International Knee Documentation Committee and Lysholm scores in all their patients at final follow-up and all patients returned preinjury activity level. Ross et al. noted one clinical failure in a case series that included five patients [12]. DiFelice et al. describe three cases of arthroscopic PCL repair with excellent Lysholm and Modified Cincinnati Knee scores at minimum two-year follow-up [13]. All three studies describe similar surgical techniques in which the PCL is sutured, passed through a femoral tunnel, and fixed to the femur on the medial cortex of the medial femoral condyle. Pisanu et al. described a similar technique in pediatric patients in which the femoral tunnels are placed in the distal part of the medial gutter, to avoid the physis [14]. Others have recently described different techniques of PCL repair, which include addressing the anterolateral and posteromedial PCL bundles individually and using suture anchors as proximal fixation [15,16].

The technique utilized in this case series is most similar to the one described by Hopper et al. [17]. DiFelice and Van der List recently described PCL repair with SA with a similar technique; however, they utilized suture anchors for proximal fixation instead of a button [16]. Additionally, Vermeijden et al. reviewed outcomes following arthroscopic PCL repair and included outcomes from their own cohort as well. In their cohort, they identified one failure out of a total of 21 MLKIs patients who underwent PCL repair with no patients developing arthrofibrosis [7].

There are several advantages of PCL repair in the setting of MLKI. There is no graft harvesting, and therefore, no risk of graft site pain or infection. In addition, there is a reduced risk of tunnel convergence with PCL repair because the procedure does not require that bone tunnels be drilled into both the distal femur and proximal tibia. PCL repair is a less invasive procedure compared to PCL reconstruction, which facilitates early rehabilitation and an easier PCL reconstruction revision surgery in the case of ligament re-rupture [17]. Indications for PCL repair are limited to proximal tears, distal tears, and avulsion injuries [7]. Contraindications include midsubstance tears or retracted tears where the PCL cannot be approximated to its attachment, both of which are recommended to be treated with PCL reconstruction [7].

In MLKI, the order of ligament tensioning has been shown to influence the final tibiofemoral orientation [18]. In this study, the PCL was tensioned before the ACL, as superior tibiofemoral orientation has been demonstrated biomechanically when the PCL is tensioned first [18]. Furthermore, the optimal timing of PCL repair surgery remains controversial. PCL reconstruction is typically performed several weeks after the initial injury to optimize ROM and reduce the risk of arthrofibrosis [7]. PCL repair is usually performed earlier than reconstruction to prevent retraction of the ligament and to minimize scarring. DiFelice and Van der List have suggested performing PCL repair within one to three weeks postinjury while the PCL tissue is optimal and there is decreased swelling and inflammation [19]. In these two cases, both PCL repairs were performed three days postinjury.

Participation in postoperative rehabilitation is an essential aspect in treating MLKIs to allow for a return to full ROM and activity [20]. Of note, patient 1 was “somewhat compliant” with physical therapy and he returned to his preinjury activity level at six months postoperatively. In contrast, patient 2 was “not at all” compliant with physical therapy and stated that he was only able to participate in his baseline activities at 50% of preinjury intensity. Additionally, patient 1 had a much higher preinjury activity level compared to that of patient 2. Patient 1 had stated that he engaged in athletic activity five or more times per week before his injury, while patient 2 had stated that he engaged in athletic activity only one to two times per week.

## Conclusions

This series of two cases of PCL repair with SA in MLKIs demonstrates that PCL repair with SA is a viable procedure that can result in excellent short-term outcomes and restore knee stability. Despite one patient developing arthrofibrosis, which necessitated manipulation under anesthesia and arthroscopic lysis of adhesions two months postoperatively, both patients had full ROM by one-year follow-up. At the two-year follow-up, both patients demonstrated improvements in KOOS that exceeded the MCID as reported in the literature for ligamentous knee injuries. Moreover, both patients returned to the sport, with one patient returning to full preinjury level of sport at six months postoperatively while the other patient returned to 50% of preinjury intensity at two-year follow-up.
